# A Case Report on Acute Fatty Liver of Pregnancy: A Difficult Differential Diagnosis of Liver Disorder

**DOI:** 10.7759/cureus.42733

**Published:** 2023-07-31

**Authors:** Shivangni Sinha, Jyotsna Yadav, Tarun Pradhan

**Affiliations:** 1 Obstetrics and Gynecology, All India Institute of Medical Sciences Patna, Patna, IND; 2 Obstetrics and Gynecology, BP Koirala Institute of Medical Sciences, Dharan, NPL; 3 Obstetrics and Gynecology, Birat Medical College and Teaching Hospital, Dharan, NPL

**Keywords:** reye’s syndrome, pancreatitis, coagulopathy, liver failure, mortality, fatty liver

## Abstract

Acute fatty liver of pregnancy is a rare but potentially dangerous pregnancy condition with significant maternal and fetal fatality rates. The disorder is driven by a complex pathophysiology and clinically manifests as a rapid worsening in health conditions, increasing the rate of mortality and necessitating expert diagnosis and management. The condition progresses from spontaneous resolution to post-operative complications, resulting in negative consequences. We offer a case report of a young primigravida patient diagnosed with acute fatty liver of pregnancy at term. The report describes the clinical course and its effect. The perinatal result, however, could not be improved due to the late diagnosis. Over the last 40 years, death rates have been dramatically lowered because of competence and a multidisciplinary approach, increasing maternal-fetal outcomes. In this scenario, time management is crucial to success.

## Introduction

The most prevalent cause of liver failure in pregnancy is acute fatty liver, also known as acute fatty transformation or acute yellow atrophy. Sheehan first described the condition in 1940 as an “acute yellow atrophy” of the liver, which was then assumed to be caused by delayed chloroform overdose [1]. In its worst form, it affects one in 10,000 people [1]. Rare but possibly lethal, with an abrupt start and rapid development. It is associated with high maternal morbidity (10%-15%) and perinatal mortality (15%-20%) [2]. Maternal and perinatal death rates have been reported to be as high as 75% and 85%, respectively [3,4]. Nulliparous women, those carrying a male fetus, and twin pregnancies are risk factors.

Patients typically present during the third trimester or the immediate post-partum period [5] with non-specific symptoms such as nausea, anorexia, unexplained stomach discomfort, and malaise. The majority of women experience a new onset of recurrent vomiting and malaise throughout their third trimester, which may raise suspicion of acute fatty liver [6]. Early detection of acute fatty liver of pregnancy (AFLP) can be challenging since it has characteristics with other prevalent illnesses such as pre-eclampsia, viral hepatitis, and cholestasis of pregnancy. A comprehensive history and physical examination, together with compatible laboratory and imaging data, rapid symptomatic treatment, prompt delivery, and supportive care, can reduce mortality [6].

We present a case report of a young patient diagnosed with acute fatty liver at the term period of gestation (POG) with a fetal perinatal prognosis. The patient survived the concomitant consequences of post-partum hemorrhage, temporary diabetes insipidus, and coagulopathy during the condition, however, had perinatal mortality. The patient was discharged on the 28th day of her delivery after appropriate care. For the publication, written informed consent was acquired.

## Case presentation

A 24-year-old primigravida at 38 weeks of gestation (POG) appeared at our emergency department in a tertiary care center with complaints of vomiting and yellowish staining of the eyes that had been present for three days. She also reported abdominal pain and malaise. The patient also complained of dizziness on several occasions. The symptoms developed gradually. There had been no history of abdominal pain, vaginal bleeding, or leaking. The patient perceived adequate fetal activity.

The patient received regular antenatal checkups at a nearby Anganwadi center and had no history of major sickness. The patient was awake, well-oriented, icteric, and had minor pedal edema on general examination. Her blood pressure was 140/80mmHg in her right arm at the level of her heart while seated, with a pulse of 84bpm that was regular in rate, rhythm, and volume. Her cardiovascular and respiratory exams came back normal. Abdominal examination revealed a single living term fetus in cephalic presentation, with a closed and uneffaced cervix. The woman was not in labor. The baseline investigations were completed, and the patient was admitted for supportive treatment and monitoring (Table 1).

**Table 1:** Baseline investigation of patient at the time of admission

Baseline Investigations	Results (Units)	Normal reference value (Units)
AST/ALT/ALP	285/310/1243 IU/L	AST: 7-56 IU/L
		ALT: 8-45 IU/L
		ALP: 32-414 IU/L
Total/conjugate bilirubin	4.6/2.5 mg/dL	Total bilirubin: 0.1-1.2 mg/dL
		Conjugate: 0-0.1 mg/dL
Total protein/albumin	6.8/3.6 mg/dL	Total protein: 6.0-8.3 g/dL
		Albumin: 3.1-5.1 mg/dL
Urea/creatinine	33/1.9 mg/dL	Urea: 3-11 mg/dL
		Creatinine: 0.4-0.8 mg/dL
RBS	36 mg/dL	80- 140 mg/dL
Hemoglobin	11.6 g%	12-16 g%
Total count	22,900/mm^3^	4,500-11,000/mm^3^
Platelet	214,000/mm^3^	199,000-244,000/mm^3^
PT/INR	30/3.12	PT: 12.6-13.26 sec
		INR: 0.8-1.2
ABG	Metabolic acidosis	pH: 7.35-7.45
		pCO_2_: 35-45 mmHg
		pO_2_: 75-100 mmHg
		HCO_3_: 22-26 mEq/L
		O2 saturation: >95%
Viral markers (Hep A, B, C, E)	Negative	Negative: viral copies not detected
		Positive: viral copies detected
		Equivocal: indeterminate
Urine RE/ME	Albumin: trace	Glucose/blood: negative
		Ketone/ Albumin: negative
		Urobilinogen/bilirubin: negative
		Leukocute esterase/nitrite: negative
		WBC/Bacteria: none

Following comprehensive counseling, the patient was induced into childbirth. Her labor was closely followed, and she underwent instrumental delivery due to fetal bradycardia. She delivered a fresh stillbirth male fetus weighing 3.15kg. She experienced primary post-partum hemorrhage, which was conservatively treated with uterotonics and balloon tamponade. The patient suffered a second bout of hemorrhage 18 hours after her delivery with the balloon tamponade in place, and she was shifted to the operation theatre (OT) for surgical intervention to control the hemorrhage. A hysterectomy was performed to save her life. She displayed signs of metabolic acidosis and coagulopathy intraoperatively. She remained intubated and was transferred to the critical care unit, where she was given broad-spectrum antibiotics with inotrope support. The patient required mechanical ventilation for eight days. During her ICU hospitalization, she got a total of 16 units of fresh blood, 20 units of fresh frozen plasma, one unit of packed cell volume, 10 pints of platelet-rich plasma, and eight units of cryoprecipitate. Her coagulopathy and hepatic dysfunction improved. Her ultrasound results were normal. She also had increased urine production during the recuperation time, which was treated conservatively. With strong supportive care, the patient recovered completely. The patient was discharged on the 28th day of her hospitalization.

## Discussion

Acute fatty liver is linked to recessively inherited mitochondrial fatty acid oxidation defects [7]. Mutations typically impact the pathway's final oxidative stages. The G1528C (60%) and E474Q (19%) mutations of the gene on chromosome 2 that codes for long-chain-3-hydroxylacyl-coA-dehydrogenase (LCHAD) are the most frequent. The other involves MCHAD and SCHAD (mid and short-chain dehydrogenase) [8]. A deficiency in mitochondrial fatty acid beta-oxidation is implicated in the pathogenesis. An individual heterozygous for enzymatic alterations in fatty acid oxidation would not have aberrant fatty oxidation under normal conditions. When a heterozygous mother carries a fetus with these enzyme homozygous mutations, fetal fatty acid accumulates and returns to the mother's circulation [8]. If the lady has a homozygous enzyme-deficient fetus, recurrence in subsequent pregnancies is likely. A survey found a 21% chance of recurrence in a future pregnancy in a subset of women who became pregnant again, with 80% of recurrent cases presenting in a milder form [9].

Acute yellow atrophy is characterized by enlarged hepatocytes with central nuclei and cytoplasm packed with microvesicular fat on gross examination. There are no symptoms of cholestasis or necrosis, and there is just minor inflammation. Additionally, peri-portal sparing is observed [10]. Endothelial cell activation is substantial at the cellular level, with capillary leakage generating hemoconcentration, hepato-renal syndrome, ascites, and pulmonary edema [10].

High-power microscopic examination of the liver reveals a dual picture of macro and microvesicular steatosis. In situations of hemoconcentration, uteroplacental insufficiency, and severe acidosis, fetal death is more common [11]. If the fetus survives, it may have a Reye-like syndrome of hepatic encephalopathy and severe hypoglycemia due to a homozygous fatty acid oxidation deficiency [11]. A retrospective analysis found that the AFLP group had a considerably greater frequency of obstetrical problems, including placental abruption (12.7%), meconium stained (II-III) (40%), and postpartum hemorrhage (52.7%) [11]. The hallmark treatment of AFLP is supportive care and pregnancy termination. Induction of labor with close fetal monitoring is the recommended route of delivery.

Because the viable fetus tolerates labor poorly, a cesarean section is also advised, which increases maternal risk in cases of severe coagulopathy. Blood products, fresh frozen plasma, cryoprecipitate, and platelets have all become critical. The liver enzymes, as well as sequelae such as encephalopathy, resolve spontaneously within seven to 10 days of intensive supportive care until complete recovery is reached [12]. During her recuperation, the patients are still in danger of getting temporary diabetes insipidus and acute pancreatitis, as in our case. Pancreatitis is a potentially fatal consequence of pregnancy-related acute fatty liver, and all patients with this diagnosis should be screened for abnormalities [13]. Critically ill patients should be moved to specialized centers as soon as possible. Plasma exchange functions as an artificial liver, eliminating toxins from the body. In this process, blood cells containing harmful compounds are removed from the patient's plasma, mixed with plasma from normal people, and returned to the patient [14]. A study conducted between 2007 and 2012 found plasma exchange with continuous renal replacement therapy (PE+CRRT) to be a safe and effective approach that should be implemented immediately at the beginning of hepatic encephalopathy and/or renal failure in AFLP patients [14]. Because of the comparable appearances of liver illnesses during pregnancy, such as pre-eclampsia, hemolysis, elevated liver enzymes, and low platelet (HELLP) syndrome, viral hepatitis, drug-induced hepatitis, and intra-hepatic obstetrics cholestasis [7,15], the diagnosis might be challenging. Pregnancy with jaundice has many differential diagnoses, but AFLP was diagnosed using the Swansea criteria, which are a collection of symptoms, signs, biochemical, and imaging findings in which the presence of six or more findings in the absence of other causes establishes a clinical diagnosis of AFLP (Table 2) [16,17]. In our case, the criteria were met, indicating that acute fatty liver was the correct diagnosis.

**Table 2:** Swansea criteria to diagnose acute fatty liver of pregnancy (AFLP)

Clinical features	Vomiting abdominal pain polydipsia/polyuria encephalopathy
Hepatic	Bilirubin > 14micromol/L, AST/ALT > 42IU/L, Ammonia > 47micr/L
Renal	Urea > 340micr/L, Creatinine > 150micr/L
Endocrine	Glucose < 4 mmol/L
Hematological	Leukocytosis > 11,000, Coagulopathy PT> 14 or APTT >34 sec
Radiological	Abdominal USG: bright liver echo texture/ ascites
Histological	Micro vesicular steatosis

In our case, she had presented with vomiting, jaundice, and malaise, and investigations revealed coagulopathy, acute kidney injury, elevated bilirubin, elevated transaminase, leukocytosis, recurrent hypoglycemia, and a normotensive patient, therefore an AFLP diagnosis was preferred. The mainstay of treatment is prompt diagnosis and delivery. In our case, the baby died as a result of the delay in diagnosing the problem. The patient was scheduled for a caesarian section due to a non-reassuring stress test, but the procedure was postponed due to coagulation failure, and blood products were administered to control the anomaly.

## Conclusions

AFLP likely occurs in the last trimester or early immediate postpartum. It is a liver disorder difficult to diagnose as it shares common clinical manifestations with viral hepatitis/ obstetrics cholestasis/ preeclampsia causing liver failure. Hence, better clinical understanding, prompt treatment, and a multidisciplinary approach can reduce mortality.
